# Toxic Anterior Segment Syndrome following Phacoemulsification Secondary to Overdose of Intracameral Gentamicin

**DOI:** 10.1155/2014/143564

**Published:** 2014-12-10

**Authors:** Yaran Koban, Selim Genc, Gorkem Bilgin, Halil Huseyin Cagatay, Metin Ekinci, Melin Gecer, Zeliha Yazar

**Affiliations:** ^1^Department of Ophthalmology, Faculty of Medicine, Kafkas University, Merkez, 36100 Kars, Turkey; ^2^Department of Ophthalmology, Dr. Lutfi Kirdar Kartal Education and Research Hospital, 34865 Istanbul, Turkey; ^3^Department of Ophthalmology, Hacettepe University Beytepe Health Center, 06800 Ankara, Turkey; ^4^Department of Pathology, Dr. Lutfi Kirdar Kartal Education and Research Hospital, 34865 Istanbul, Turkey; ^5^Kafkas University, 36100 Kars, Turkey

## Abstract

*Objective*. To report a case of toxic anterior segment syndrome (TASS) that was caused by inadvertent anterior chamber and cornea stromal injection with high dose gentamicin following cataract surgery. *Methods*. Case report. *Results*. We report a 72-year-old female patient who developed TASS that was caused by high dose gentamicin (20 mg/0.5 mL), which was inadvertently used during the formation of the anterior chamber and hydration of the corneal incision. Unlike previous cases, hyphema and hemorrhagic fibrinous reaction were seen in the anterior chamber. Despite treatment, bullous keratopathy developed and penetrating keratoplasty was performed. The excised corneal button was sent for histopathological examination. *Conclusions*. Subconjunctival gentamicin is highly toxic to the corneal endothelium and anterior chamber structures. Including it on the surgical table carries a potentially serious risk for contamination of the anterior chamber.

## 1. Introduction

Toxic anterior segment syndrome (TASS) is an acute sterile anterior segment inflammation that develops after anterior segment surgery. It typically presents within 12 to 48 hours of surgery [1]. Its hallmarks are minimal or no pain, pronounced cellular and fibrinous anterior chamber reaction, and diffuse limbus-to-limbus corneal edema secondary to damage from a toxic insult to the endothelial cell layer without posterior segment involvement [2]. Various entities have been shown to cause TASS, including improperly mixed or dosed intracameral anesthetics and antibiotics, endotoxin, incorrectly balanced salt solution (BSS), pH, and/or osmolarity, as well as sterilization substances left on surgical instruments [3–6]. We describe a case of TASS that is linked to the intracameral injection of a subconjunctival dosage (40 mg/mL) of gentamicin at the end of a routine cataract surgery; this is the first reported human case with a hyphema and hemorrhagic fibrinous reaction during the course of TASS.

## 2. Case Report

A 72-year-old woman patient with 2-year history of reduced vision in the left eye was admitted to our department. Her best-corrected spectacle visual acuity (BCVA) was 20/60 OD and 20/200 OS. Intraocular pressure (IOP) was measured as 15 mmHg OU. Slit-lamp examination results were unremarkable except for a grade 2 nuclear sclerotic cataract (NO2, NC2, according to Lens Opacities Classification System III) in the right eye and a grade 4 nuclear sclerotic cataract (NO4, NC4, according to Lens Opacities Classification System III) in the left eye [7]. The patient's left eye underwent an uneventful clear corneal phacoemulsification through clear corneal incision with implantation of a hydrophobic acrylic intraocular lens (IOL) under topical anesthesia. The Series 20,000 Legacy phacoemulsification unit (Alcon Laboratories) was used in all cases, with a flow rate of 36 mL/minute, maximum ultrasound power of 65%, and a phaco tip angled at 30 degrees. On the day of that particular surgery, all of the cataract surgeries were performed by one surgeon in the same operating room. However, two nurses were shifting between the procedures.

At the end of the operation, the nurse in charge of surgical assistance inadvertently gave to the surgeon 40 mg/mL of a preservative-free aqueous solution of gentamicin, which had been prepared for subconjunctival injection, instead of BSS. As a result, an almost 2 mL gentamicin (40 mg/mL) was used for the formation of the anterior chamber and for hydration of the corneal incision. The surgeon recognized the erroneous injection immediately and applied anterior chamber hydration. The operation was ended with the administration of 1 cc 0.05% moxifloxacin to the anterior chamber. Postoperatively, ofloxacin 0.3% eye drop 4 times a day, prednisolone acetate 1% eye drop once hourly, and ciprofloxacin ophthalmic ointment once a day were started.

One hour after the operation the patient had mild conjunctival chemosis, limbus-to-limbus corneal edema, and generalized Descemet's membrane folds without pain. The pupil was middilated, irregular, and unresponsive to light. Grade 1 hyphema with hemorrhagic fibrinous reaction was observed in the anterior chamber. There was no hypopyon. IOP was 17 mmHg and it did not show an increase during the follow-up. Topical prednisolone acetate 1% hourly, cyclopentolate hydrochloride 1% 3 times daily, NaCl 5% 6 times daily, ofloxacin 0.3% 4 times daily, ciprofloxacin ophthalmic ointment, and oral prednisolone 0.5 mg/kg once daily were started. On postoperative day 1, the patient had an intense corneal edema and visual acuity was measured as finger counting from 1 meter. However, there was no hyphema and hemorrhagic fibrinous reaction was decreased; by postoperative day 3 fibrin formation in the anterior chamber had totally disappeared. The fundus could not be seen due to severe corneal edema following cataract surgery. Results of B-scan ultrasonography were normal.

**Figure 1:**
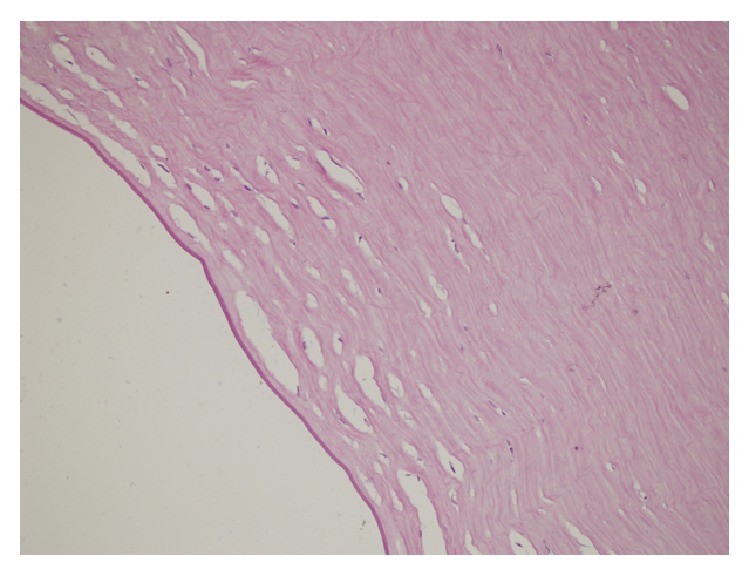
Light microscopy of stroma and Descemet's membrane (high magnification ×400) showing cystic spaces in stroma, Descemet's membrane irregularities, and complete absence of endothelial layer. 180 × 135 mm (300 × 300 DPI).

**Figure 2:**
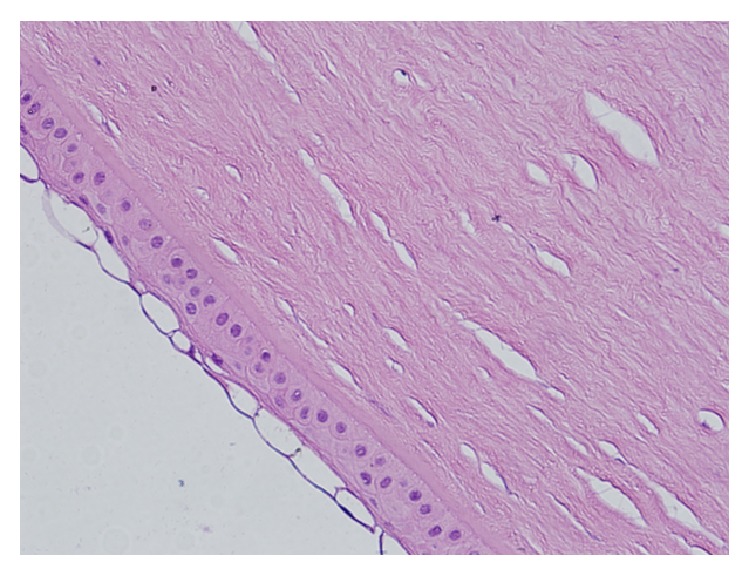
The image of the same corneal specimen at high magnification (×400). The thin-walled cystic vesicles are seen on the outer (epithelial) surface of the cornea. 180 × 135 mm (300 × 300 DPI).

During the follow-up the folds of Descemet's membrane improved. However, corneal edema did not regress, bullous keratopathy developed, and the patient needed penetrating keratoplasty. Following the keratoplasty, histopathological examination of the excised corneal button showed a vacuolated and thinned epithelial cell layer, disturbed collagen bonds, Descemet's membrane irregularities, and endothelial cell layer loss (Figures 1 and 2).

After penetrating keratoplasty, the patient developed a fibrous membrane adherent to the lens implant and iris 2 days postoperatively. Visual acuity was counting fingers at 3 meters. IOP was normal. Topical steroid eye drops (prednisolone acetate 1% eye drop once hourly) were given. After 4 days, the membrane started to reduce from upper and lower margins of the pupil. Visual acuity was 20/200 at 1 month, 20/100 at 2 months, and 20/60 at one year. Because the membrane did not disappear totally, excision of the fibrous anterior chamber membrane was planned. However, the patient did not accept another surgery. She was satisfied with her vision.

At the end of the first-year follow-up, BCVA and IOP were measured as 20/60 and 13 mmHg, respectively. The corneal graft was transparent. The pupil was irregular, fix-dilated, and unresponsive to light, while transillumination defects in the iris and persistent pupillary membrane were present. On ophthalmic examination, fundoscopic findings were unremarkable and normal macular thickness was determined by optical coherence tomography.

## 3. Discussion

TASS is a rare anterior segment syndrome which can cause serious endothelial damage. Avisar and Weinberg reported a reduction both in endothelial cell number and in the ratio of hexagonal cells in cases with TASS [2]. Arslan et al. demonstrated that the endothelial layer completely disappeared histopathologically in patients with TASS [3]. While the corneal edema in early TASS is the result of the destruction of the linkages between endothelial cells, the edema in the later stages is caused by apoptosis of the endothelial cells [4].

The clinical course is associated with the content of the toxic agent, contact time, and the time when treatment began. In moderate cases, inflammation may decrease rapidly and corneal edema may regress within 1–3 weeks. Corneal recovery in patients with moderate syndrome may take 6 weeks and mild corneal edema may be permanent. In severe cases, corneal edema may be permanent and may result in bullous keratopathy. If corneal edema does not regress within 6 weeks, it should not be expected to regress later, and penetrating keratoplasty should be considered. In moderate cases, IOP can be brought under control with drug therapy. However, in severe cases, the IOP cannot be controlled with medical treatment and surgical treatment is usually required. Middilated pupil unresponsive to light reflex may develop due to iris damage, and this may be permanent [4].

In our case, corneal edema did not regress, bullous keratopathy developed, and penetrating keratoplasty was needed. Histopathological examination of the corneal specimen showed a vacuolated and thinned epithelial cell layer, disturbed collagen bonds, Descemet's membrane irregularities, and endothelial cell layer loss (Figures 1 and 2).

Our case is distinct from the previously reported cases in that this is the first human case of TASS with hyphema and iris hemorrhage. Leder et al. observed a TASS-like response in rabbit eyes that was caused by enzymatic cleaners used for ophthalmic surgical instruments [5]. At the end of that study, the hyphema that started within the first hour and dissolved within 72 hours at the latest, together with iris vessel injection, was observed in some of the rabbit eyes. They suggested that the reason hyphema and iris hemorrhage had not been reported in humans before was that the patients had not been seen by an ophthalmologist up to 24 hours postoperatively. The hyphema and iris hemorrhage in our patient can be explained by exposure to the highly toxic agent and evaluation of the patient by the ophthalmologist in the early postoperative period.

Kobayakawa et al. reported severe damage to rabbit corneal endothelial cells caused by gentamicin ≥ 2 mg/mL, which is 20 times diluted when compared to the original solution of 40 mg/mL and 10 times diluted when compared to 20 mg/mL, which is the concentration commonly used for subconjunctival gentamicin injection [6]. Although subconjunctival injection is currently accepted as a safe technique, 2 cases of TASS associated with the intraocular spillover of gentamicin after subconjunctival administration at the end of routine cataract surgery have been reported [8]. At the same time, as in our case, its presence at the operating table with other fluids used in the anterior chamber also carries a risk in terms of contamination of the anterior chamber with subconjunctival gentamicin. Ha et al. reported a case of inadvertent anterior chamber and cornea stromal injection with high dose gentamicin and dexamethasone during cataract operation [9]. We believe that our case is the second report dealing with endothelial toxicity in humans after anterior chamber injection of high dose gentamicin.

## 4. Conclusion

We suggest that our case is admonitory for the dangers of subconjunctival gentamicin during ophthalmic surgeries. Surgeons should be very careful while administering medications to the anterior chamber, alert for the danger of errors in preparing diluted solutions and surgical staff should be educated about this topic. To prevent such a mix-up in the operating room, different types of syringes, such as insulin syringes, should be used for anesthetics and antibiotics prepared for subconjunctival injection. Because the subconjunctival preparations' place on the surgical table carries a potentially serious risk in terms of the contamination of anterior chamber, they could be prepared at the end of the operation.
